# Cross-cultural adaptation and validation of Spanish version of The Foot and Ankle Ability Measures (FAAM-Sp)

**DOI:** 10.1186/s13047-017-0221-6

**Published:** 2017-08-22

**Authors:** Pablo Cervera-Garvi, Ana Belen Ortega-Avila, Jose Miguel Morales-Asencio, Jose Antonio Cervera-Marin, Rob Roy Martin, Gabriel Gijon-Nogueron

**Affiliations:** 1 Nursing and Podiatry, University of Malaga, C/Arquitecto Francisco Peñalosa Ampliación Campus Teatinos, 29071 Málaga, Spain; 2 Duquesne University Physical Therapy, 600 Forbes Avenue, 111A RSHS, Pittsburgh, PA 15282 USA

**Keywords:** Foot, Ankle, Questionnaire, Cross cultural adaptation

## Abstract

**Background:**

The Foot and Ankle Ability Measure (FAAM) is a Patient Reported Outcome (PRO) commonly used to determine the effectiveness of therapeutic interventions for patients with foot and ankle pathologies and associated impairments of body function and structure, activity limitations, and participation restrictions. The aim of this study was to cross-culturally adapt the FAAM into Spanish.

**Methods:**

Cross-cultural adaptation was performed according to the international guidelines of the International Society for Pharmacoeconomics and Outcomes Research. Cronbach’s alpha, test re-test reliability, and item-total and inter-item correlations were analyzed. Confirmatory factor analysis (CFA) was carried out to test construct validity. Pearson correlations were calculated to assess the convergent validity between FAAM and EuroQol-5.

**Results:**

Spanish data set comprised 194 patients, with a mean age of 38.45 (16.04) and 130 (67.1%) were female, seeing a podiatrist with a wide variety of foot and ankle related disorders. CFA was carried out to test structure matrix (which has three factors). The test–retest reliability was high with global ICC of 0.95 (95% CI: 0.93 to 0.98). A 15 items version of the FAAM-Sp Activities of Daily Living (ADL) obtained the best fit: relative chi-square (*x* ^2^/df) of 2.46, GFI 0.90 CFI 0.95, NFI 0.93, and RMSEA 0.08 (90% CI 0.04 to 0.09). For exploratory factor analysis for the FAAM-Sp Sport, a one factor solution was obtained, which explained 76.70% of total variance. CFA corroborated this model with an excellent goodness of fit:: relative chi-square (*x* ^2^/df) of 0.80, GFI 0.99 CFI 1.00, NFI 0.99, and RMSEA 0.00 (90% CI 0.00 to 0.75).

**Conclusions:**

This study validated a new 15-item FAAM-Sp ADL and FAAM-Sp Sport subscales, which can be used as a self-reported outcome measure in clinical practice and research for patients resident in Spain whose main language is Spanish.

## Background

The Foot and Ankle Ability Measure (FAAM) is an instrument to evaluate, from a self-reported perspective, the physical function and activities of daily living for individuals with foot and ankle related impairments. It is commonly used to assess the effectiveness of therapeutic interventions for patients with foot and ankle pathologies and associated impairments of body function and structure, activity limitations, and participation restrictions [1].

Instruments that evaluate patient reported outcomes (PROs) should contain items that assess an individual’s body function and structure, activity limitations, and participation restrictions [1, 2]. Therefore, an instrument should have an appropriate conceptual framework, with items clearly defined and evidence of validity, reliability, and responsiveness to change [3].

The FAAM consists of 29 items, divided into two subscales: (i) activities of daily life (ADL), measured by 21 items, and, (ii), sport activities, assessed by eight items [1]. The original FAAM was developed for an English-speaking population and was found to have evidence of reliability, validity, and responsiveness in patients with a wide range of foot and ankle related pathologies [4]. Additional evidence of its validity has been provided for specific populations, including athletes with chronic ankle instability [5] and individuals with diabetes mellitus [4]. The FAAM has been translated into Persian [5], French [6], German [7], Japanese [8], Brazilian [9], Thai [10], Turkish [11], Chinese [12] and Dutch [13] languages. Although the FAAM is widely used, to our knowledge it has not been adapted and validated for Spanish speaking populations.

Currently there is a lack of evidence to support the use of FAAM for Spanish speaking patients with musculoskeletal foot and ankle disorders. The aims of this study were twofold: (i) to carry out a cross-cultural adaptation of the FAAM into Spanish, (ii) to determine the psychometric properties of the Spanish version of the FAAM (FAAM-Sp).

## Methods

The study consisted of 2 phases: 1) cross-cultural adaptation of the original English FAAM to produce FAAM-Sp and 2) providing evidence of validity for the FAAM-Sp by examining its psychometric properties and conducting a confirmatory factor analysis. Ethical approval was obtained from the Ethics Committee of the University of Malaga, Spain. All participants in the study gave a written informed consent. Participants were recruited from seven podiatry clinics participating in the Private Podiatry Clinics Network located in the south of Spain. Participating patients met the following inclusion criteria: (1) aged 18 or over, (2) required podiatry treatment, (3) were in current employment (4) were mobile and capable of walking household distances unaided, (5) were native Spanish speakers. Participants were excluded from the study if they were unable to read.

### Cross-cultural adaptation

**Fig. 1:**
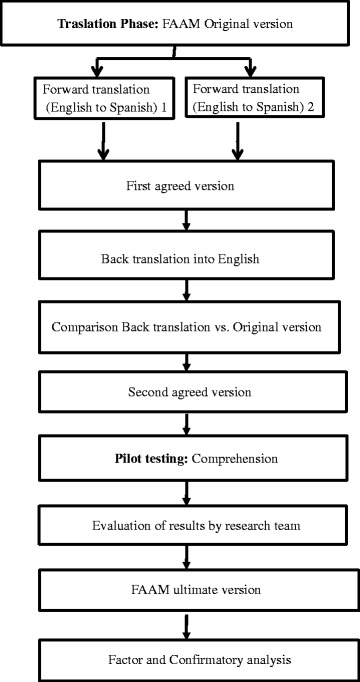
Cross-cultural adaptation process

The cross-cultural adaptation process was undertaken using the guidelines derived from the methodology recommended by the International Society for Pharmacoeconomics and Outcomes Research (ISPOR) for the translation and validation of patients reported outcome measures [14]. Cross-cultural adaptation involved eight stages: (1) forward translation, (2) reconciliation; (3) back translation, (4) back translation review, (5) harmonisation, (6) pilot testing/cognitive debriefing, (7) pilot testing review/review of cognitive debriefing results and (8) proof reading. Fig. 1 summarises the cross-cultural adaptation process.

### Forward translation

Two forward translations in Spanish were undertaken from the original English version of the FAAM. The translations were undertaken by two independent health professionals who were native residents of Spain and fluent in both Spanish and English. Both translators based their translations on the guidelines provided by ISIS outcomes for the translation and linguistic validation process [15].

### Forward translation reconciliation

The two forward translations were reconciled into one version (draft 1) by two original translators, a third independent translator and feed-back from the principal researcher in case of doubts [GGN].

### Back translation

The reconciled Spanish language version (draft 1) was back translated into English independently by two professional English native translators resident in Spain. The translators had no prior knowledge of the FAAM and were not given the original wording of the English version of the FAAM.

### Back translation review

The project lead [GGN] and a native Spanish speaker resident in UK and fluent in both languages reviewed the back translation for any discrepancies in meaning or terminology used. Any problematic item was discussed until the discrepancies were resolved. This process resulted in a refined second draft of the Spanish translation (draft 2).

### Harmonisation

To produce the final Spanish language translation, a harmonisation meeting was undertaken involving three Spanish translators, the project lead [GGN] and the developer of the original USA version of the FAAM [RRM]. During this meeting, any discrepancies or issues that were highlighted from the back translation were discussed, the translated version of the FAAM was evaluated and a final version agreed.

### Pilot testing/cognitive debriefing

Once the translation process was completed, the translation was formatted to match precisely the original American language version. The translated FAAM version was initially assessed for comprehensibility in five patient participants, who were Spanish residents and native speakers, met the inclusion criteria described above and had a low educational background without being illiterate. At this stage, each participant was asked by the in-country investigator [PCG] to carry out the following tasks:

- To complete a copy of the translated FAAM and time needed.
- To comment on the response options within the back-translated FAAM.
- To comment on any wording that was difficult to understand.
- To suggest alternative wording/phrasing for any wording that was difficult to understand.
- To describe in their own words what the wording meant to them. These responses were recorded verbatim and translated into English. The five patients’ responses were summarised by the project lead [GGN]. This summary also contained any changes, recommendations or suggestions indicated by the participants and in-country investigators.

### Pilot testing review/review of cognitive debriefing results

To improve the performance of the translated questionnaire, the pilot testing results were reviewed by the in-country investigators [PCG]. At this stage, any item that caused comprehensibility difficulties for more of the 40% of the participants was reviewed, and any modifications suggested by the respondent’s comments were incorporate to the final translated version.

### Proof-reading

The project lead [GGN] and a translator [ABOA], who was not involved in the translation process previously, independently proofread the final formatted translation, and any suggested changes were discussed with the project lead [GGN]. Furthermore, the Flesh Reading Ease test and the Flesh Kincaid Grade Level were calculated for readability [16].

Following this process, a final draft of the FAAM translated and culturally adapted into ‘Spanish for Spain’ was finalized and entered into the cross-cultural validation phase (final draft).

### Evidence of validity

Before completing the questionnaire, the following data for each participant were recorded: age, sex, professional status, education level, affected extremity and diagnosis. Podiatrists working in the seven different private clinics of podiatry collected the questionnaires. They addressed any possible concern of the participants. Items were numbered from 1 to 29. Items 1 to 21 were from Activities of Daily Living (ADL) subscale and items 22 to 29 were from the Sports subscale. Each question is rated on a scale of 0 to 4, with 0 being “unable to do” and 4 being “no difficulty”. None answer responses are not counted. Thus, each subscale has a maximum potential score (84 ADL and 32 Sport subscales). The score for each subscale is divided by the maximum potential score of the subscale and multiplied by 100 to get a percentage, with a higher score meaning a higher level of function on each of the two subscales.

### Sample size calculation

To test a two-factor model, assuming the null hypothesis of a mean square error of approximation (RMSEA) from 0.04 to 0.085, with an alpha value of 0.05, a statistical power of 0.8 and a maximum of 26 degrees of freedom, as suggested by MacCallum et al. [17], a sample of 196 participants was required which was over-estimated by 10% to cover possible losses. The calculations were performed with the Statistica 12 software [18].

### Data analysis

Descriptive statistics were carried out with means, standard deviations, and absolute and relative frequencies. Analysis of normality of distributions was evaluated by the Kolmogorov-Smirnov test, symmetry analysis and kurtosis. Internal consistency was calculated using Cronbach’s alpha. A Cronbach’s alpha between 0.70 and 0.95 was considered “good” [19]. Moreover, item-total and inter-item correlations were assessed. Pearson correlations were calculated to assess the convergent validity between FAAM and EuroQol-5D Spanish version [20]. Content validity was assessed by the distribution of the scores and occurrence of floor-ceiling effect. The floor-ceiling effect was estimated through the endorsement frequency, with a maximum accepted value of 85%. To assess factor structure, a confirmatory factor analysis was performed, the evaluated model was fit with the following parameters: the penalizing function (chi square/ df), which is indicative of good fit with values less than 3; Root Mean Square Error of Approximation (RMSEA) and confidence intervals (CI 90%), taking the value 0.05 as cut-off of good fit; Normed Fit Index (NFI), the Comparative Fit Index (CFI), and Goodness of Fit Index (GFI) with a minimum value of good fit of 0.90. Multinormality was evaluated with the Mardia’s coefficient (multivariate curtosis), which could not be over “p” (p + 2), where “p” are the number of observed variables [21].

Reliability was performed using the Intraclass Correlation Coefficient Type 2.1 (ICC 2.1) [22] test-retest methodology in the full sample recorded at baseline and 1 week (7 days) following a period of no treatment.

All the analyses were performed with SPSS 23 [23] and AMOS 21 [24].

## Results

### Cross-cultural adaptation

The FAAM was translated into Spanish and culturally adapted to provide the new FAAM-Sp (Fig. 1). In the pilot testing phase, results showed no discrepancies in meaning or terminology used in the translated version of the FAAM. Hence, no modification of this version was performed. Participants did not request assistance in interpretation of the questionnaire or any of its items. The time needed to fill out the questionnaire was 3.83 min (SD 0.99). The result for Flesh Reading Ease test was 56.4 and 7.8 for the Flesh Kincaid Grade Level.

Items 5, 9, 12, 15, 16, 17 and 21 showed backup frequencies between 85.4 and 89.6%.

### Construct validity

A total number of 204 participants were included in this study. However, 10 participants were excluded from data analysis because they did not fully complete the FAAM-Sp. Hence, 194 participants (130 women and 64 men), with a mean age of 38.45 (SD: 16.04) were assessed. The characteristics of the sample are presented in Table 1.

**Table 1:** Characteristics of participants

	(*n* = 194)	
Age (years) Mean (SD)		38.45 (16.04)
Professional status n (%)	Employed	95 (48.97%)
	Unemployed	25 (12.89%)
	Students	34 (17.52%)
	Retired	15 (7.73%)
	House-wife	25 (12.89%)
Educational level n (%)	Low	71 (36.6%)
	Medium	80 (41.24%)
	High	43 (22.16%)
EQ5D VAS Quality of Life. Mean (SD)		7.85 (1.53)
Pain localizations n (%)	Heel pain	49 (25.26%)
	Midfoot Pain	18 (9.28%)
	Forefoot pain	105 (54.12%)
	Toes pain	22 (11,34%)
Diagnosis n (%)	Fasciitis	45 (23.2%)
	Stress fracture	7 (3.61%)
	Achilles tendinopathy	4 (2.06%)
	Tendinopathy	6 (3.09%)
	Morton’s neuroma	19 (9.79%)
	Bunion	11 (5.67%)
	Hammer toes	13 (6.7%)
	Osteoarthrosis	21 (10.82%)
	Callus and corns	68 (35.06%)

### FAAM-Sp ADL subscale

For exploratory factor analysis, the correlation matrix for the FAAM-Sp ADL was determined as suitable from the Kaiser–Meyer–Olkin values (0.879) and Barlett’s test of sphericity (*p* < 0.001). A three factor solution was obtained, after a principal axis factor extraction and oblique rotation, with item loading (Table 2). A confirmatory factor analysis (CFA) was then carried out to test this three-factor structure. The 21 items solution did not obtain a good fit (*x* ^2^/df 4.28, *p* < 0.001, GFI 0.76, NFI 0.85, CFI 0.88, RMSEA 0.13 IC 90%: 0.12 to 0.14).

**Table 2:** Initial factor matrix for the 21-item ADL FAAM-Sp

	Factor		
	Factor 1	Factor 2	Factor 3
FAAM8	.866		
FAAM4	.857		
FAAM6	.791		
FAAM10	.768		
FAAM11	.723		
FAAM3	.684		
FAAM1	.675		
FAAM16		−.906	
FAAM18		−.859	
FAAM17		−.849	
FAAM21		−.809	
FAAM19		−.773	
FAAM20		−.761	
FAAM14			−.980
FAAM13			−.907
FAAM2			−.874
FAAM15			−.860
FAAM12			−.824
FAAM7			−.813
FAAM5			−.798
FAAM9			−.796

**Fig. 2:**
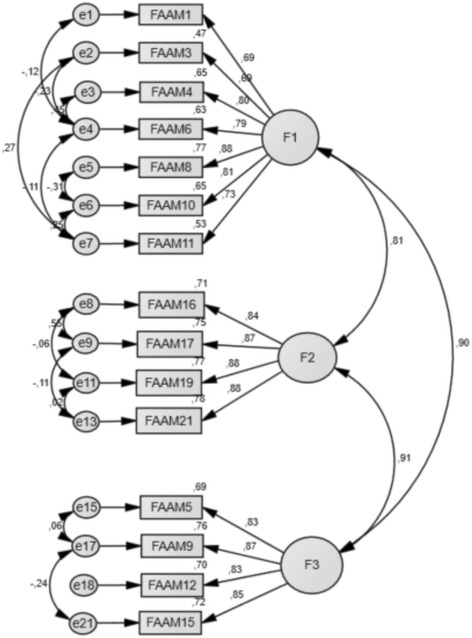
Confirmatory factor structure of FAAM-Sp ADL. F1: Factor 1; F2: Factor 2; F3: Factor 3

An additional model of 15 items was tested, after deleting items which have had a bad adjustment in the model, evaluated through their standardized residual covariances (items 2, 7, 13, 14, 18, and 20). This model obtained the best fit: relative chi-square (*x* ^2^/df) of 2.46, GFI 0.90, CFI 0.95, NFI 0.93, and RMSEA 0.08 (90% CI: 0.04 to 0.09), and fulfilled the multinormality criteria (Fig. 2).

### The FAAM sport subscale

This instrument obtained a Cronbach’s alpha of 0.95, with an inter-item mean correlation of 0.73 (Range: 0.57 to 0.88). The mean scoring was 11.59 (SD: 5.98). Item-total correlation was above 0.70 in all the items.

**Fig. 3:**
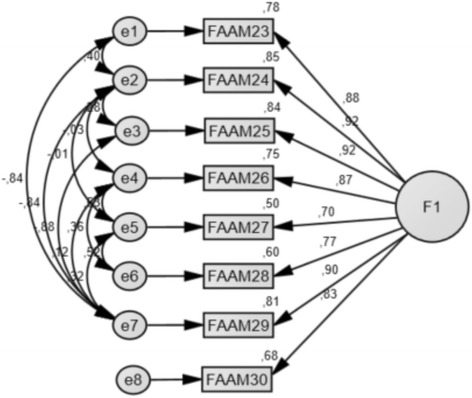
FAAM-Sp Sport structure

For exploratory factor analysis, the correlation matrix for the FAAMSp Sport was determined as suitable from the Kaiser–Meyer–Olkin values (0.905) and Barlett’s test of sphericity (*p* < 0.001). A one factor solution was obtained, which explained 76.70% of total variance. CFA corroborated this model with an excellent goodness of fit: relative chi-square (*x* ^2^/df) of 0.80, GFI 0.99, CFI 1.00, NFI 0.99, and RMSEA 0.00 (90% CI: 0.00 to 0.75), and fulfilled the multinormality criteria (Fig. 3).

No floor-ceiling effect was observed. Only items 27, 28 and 29 obtained high endorsement frequencies in the lowest value, but under 85%.

### Internal consistency

This version of the 15 items FAAM-Sp ADL showed a good degree of internal consistency for each factor (Cronbach’s alpha 0.90, 0.92 and 0.90 respectively, and 0.95 overall).

The inter-item correlation matrix is detailed in Table 3. Mean Inter-item correlation was 0.60 (range 0.44 to 0.80), 0.77 (0.72 to 0.88), and 0.71 (0.66 to 0.74), for factor 1, 2, and 3, respectively. Item-total correlation was 0.65 (Table 4). Mean values and standard deviations of each factor were: 10.04 (4.60) for factor 1, 4.81 (2.03) for factor 2 and 4.85 (2.06) for factor 3.

**Table 3:** Item-item correlations matrix for the 15-item FAAM ADL-Sp

	FAAM1	FAAM3	FAAM4	FAAM6	FAAM8	FAAM10	FAAM11	FAAM16	FAAM17	FAAM19	FAAM21	FAAM5	FAAM9	FAAM12	FAAM15
FAAM1	1.000														
FAAM3	.453	1.000													
FAAM4	.599	.551	1.000												
FAAM6	.512	.442	.797	1.000											
FAAM8	.551	.630	.731	.702	1.000										
FAAM10	.599	.539	.629	.615	.620	1.000									
FAAM11	.532	.633	.574	.517	.639	.692	1.000								
FAAM16	.465	.483	.466	.514	.523	.623	.556	1.000							
FAAM17	.436	.452	.517	.533	.587	.590	.590	.876	1.000						
FAAM19	.563	.488	.510	.577	.625	.630	.593	.720	.759	1.000					
FAAM21	.462	.463	.520	.571	.606	.630	.578	.741	.736	.778	1.000				
FAAM5	.529	.589	.631	.649	.731	.592	.501	.638	.693	.634	.601	1.000			
FAAM9	.495	.510	.639	.674	.710	.654	.581	.675	.646	.662	.702	.743	1.000		
FAAM12	.529	.523	.520	.576	.624	.623	.567	.672	.645	.684	.698	.657	.735	1.000	
FAAM15	.547	.460	.577	.579	.639	.605	.518	.625	.708	.690	.693	.713	.679	.718	1.000

**Table 4:** Inter-item correlation for each factor of the 15-item ADL FAMM-Sp

	Item-total correlation inside the factor	Square multiple correlation	Alpha if ítem deleted
FACTOR 1			
FAAM1	.653	.453	.901
FAAM3	.660	.504	.904
FAAM4	.792	.726	.887
FAAM6	.727	.685	.895
FAAM8	.797	.668	.885
FAAM10	.754	.608	.891
FAAM11	.745	.599	.891
FACTOR 2			
FAAM16	.844	.788	.897
FAAM17	.857	.803	.890
FAAM19	.818	.681	.900
FAAM21	.814	.675	.912
FACTOR 3			
FAAM5	.785	.635	.876
FAAM9	.803	.664	.870
FAAM12	.782	.631	.879
FAAM15	.782	.623	.879

Floor effect was observed in items 5, 9, 12, 15, 16, 17, and 21, with endorsement frequencies between 85.4 and 89.6%.

### Convergent validity

Statistical analyses revealed correlation coefficients of − 0.596 between the ADL subscale and the EuroQol5D (*p* < 0.001) and − 0.472 between the Sports subscale and the EuroQol5D (*p* < 0.001).

### Test re-test validity

The FAAM-Sp ADL sub-scale, showed a high test–retest reliability, with a global ICC of 0.95 (95% CI: 0.93 to 0.98). For the **FAAM-sp. Sport sub-scale,** the test-retest reliability showed an ICC of 0.97 (95% CI: 0.95 to 0.98).

## Discussion

The aims of this study were to carry out a cross-cultural adaptation of the FAAM into Spanish and to determine its psychometric properties. The results provide evidence to support the use of the FAAM-Sp for patients resident in Spain whose main language is Spanish with a wide range of foot and ankle related disorders. The original version [4] was successfully translated and cross-cultural adapted. The participants of this study had no difficulties understanding the questionnaire during the pilot study, and the translated version demonstrated good results in readability tests. A 15 item FAAM-Sp ADL and eight item Sports subscales were found to have appropriate factor structure, internal consistency, test re-test reliability, item-total and inter-item correlations, and convergent validity to the EuroQol-5D.

The sample of this study was more heterogeneous than that of previous studies, because data were collected in a podiatry clinic, with a diverse range of clinical situations with a diverse range of clinical situations (see Table 1). Nevertheless, although a great proportion were patients with callus and/or corn (35,06%), the second most frequent group of patients had plantar fasciitis or tendinopathy (28.35%), such as in previous studies [25, 26]. This is an asset from the point of view of external validity of the results, and that the FAAM could be used in patients who commonly frequent podiatry clinics, although FAAM-Sp should be validated in a specific sample of patients with ankle sprains, as this was not done in the present study.

Overall, the psychometric properties that were obtained for the FAAM-Sp ADL and Sports subscales (29 items) in the present study were similar to the original version [27]. The FAAM-Sp demonstrated excellent internal consistency, with Cronbach’s alpha values of 0.90, 0.92 and 0.90 in the three factors identified, and 0.95 overall and 0.95 was found for ADL and Sports subscales. The internal consistency of the FAAM-Sp was similar to other studies carrying out cross-cultural adaptations, such as Persian (ADL: 0.97; Sport: 0.94) [5], French (0.97 in both subscales) [6], Japanese (ADL: 0.99; Sport: 0.98) [8], Brazilian (ADL: 0.93; Sport: 0.90) [9] and Thai (ADL: 0.94; Sport: 0.88) [10].

Regarding the factor analysis of FAAM-Sp ADL, three factors were identified, and this is not consistent with the original, French [6], German [7], Persian [5], and Japanese [8] versions. The three factors that were found after this analysis, are referred to these three potential dimensions: the effort factor with the items number 1, 3, 4, 6, 8, 10 and 11; the daily activities carrying out factor 16, 17, 18, 19, 20 and 21, so the less effort made by the patient factor with items 2, 5, 7, 9, 12, 13, 14 and 15.

Factors 1 and 2 did correlate negatively, while factors 2 and 3 were found to correlate positively. The FAAM-Sp Sport subscale analysis did agree with the original version [27], and their successive adaptations such as FAAM Turkish, with an inter-item mean correlation of 0.73 [11].

For the analysis of convergent validity, the FAAM-Sp was compared to the EuroQol5D. The level of correlation was good between the EuroQol5D and FAAM-Sp ADL (− 0.596) and sport (− 0.472). The original FAAM and other versions, such as FAAM-Japanese [8], FAAM-Brazilian [9] and the FAAM-French [6], used SF36 questionnaire with a good correlation. However, in the original study of the English version of the FAAM, the analysis of convergent validity with the SF36 questionnaire presented a strong relationship in the physical function component (*r* = 0.84 and *r* = 0.78 for ADL and Sport, respectively), while the correlation with mental function was appropriately weak (*r* = 0.18 and *r* = 0.11 for ADL and Sport, respectively).

A novelty in the statistical analysis of FAAM-Sp ADL was a Confirmatory Factor Analysis (CFA) [28], in order to assess factor structure. This analysis identified the FAAM-Sp ADL subscale represented by three factors. This new approach differs to that used for the original version, which identified only one factor for the FAAM subscale ADL. Once the six items with poor model fit were removed, the 15-item FAAM-Sp obtained a good fit in CFA, since a 2.46 index of chi-square goodness of fit test was obtained [29]. In addition, a RMSEA of 0.08 (90% CI) is an indicator of a good adjustment between the measurement model and data structure [30] and a NFI of 0.93 and a CFI of 0.95 are indicators of good adjustment [31]. Our results imply that a new 15-item FAAM-Sp ADL subscale could be used by researchers and practitioners as a measuring tool for the quality of life, in patients with potential foot and ankle disorders.

This study has a number of strengths. This adapted version could be the benchmark for other FAAM transcultural studies in other Spanish-speaking populations or about specific pathologies that will determine the differences between participants, serving as screening in the clinical practise. Another contribution from this study to advancement of the field is the FAAM-Sp Sport. There are currently no questionnaires that evaluate foot function using questionnaires for patients performing sport activities, and that are sensitive to small changes, typical of those found in athletes [32].

This study presents some limitations that should be recognized. First, convenience sampling was used, and patients were recruited from private clinics only, as the Spanish public health system does not offer podiatry services. The sample used in this current study does represent patients from seven clinics, and includes patients with an appropriate range of age, educational background, and employment status.

Second, since the FAAM has been validated at the test level, it may not be possible to make cross-cultural comparisons of the foot disability at the individual item level.

In some items a ‘floor’ effect was noted. It is possible that this could be due to those participants who had foot problems that caused poor foot function. Although none of those items obtained a floor effect over 90% it would be useful to investigate this further in larger participant samples with more diverse functionality.

These findings should be checked in another sample. Further research would be needed to assess responsiveness of this new FAAM-Sp in other different foot disorders. Lastly, since this study has validated a new Spanish version of the FAAM, further cross-cultural validation would be required if this questionnaire is used in other Spanish-speaking countries, such as those in South America.

## Conclusion

This study carried out a cross-cultural adaptation and validation of Spanish language version of the FAAM ADL and Sport for Spanish population (FAAM-Sp), and an examination of the psychometric properties of this new version. Nevertheless, further studies would be useful that focused on investigation of specific patient classifications to confirm its psychometric properties.

## Abbreviations

CFA: Confirmatory factor analysis
CFI: The comparative fit index
CI: Confidence intervals
FAAM: The foot and ankle ability measures
GFI: Goodness of fit index
NFI: Normed fit index
PRO: Patient reported outcome
RMSEA: Root mean square error of approximation
